# Dietary Advanced Glycation End-Products and Colorectal Cancer Risk in the European Prospective Investigation into Cancer and Nutrition (EPIC) Study

**DOI:** 10.3390/nu13093132

**Published:** 2021-09-08

**Authors:** Elom K. Aglago, Ana-Lucia Mayén, Viktoria Knaze, Heinz Freisling, Veronika Fedirko, David J. Hughes, Li Jiao, Anne Kirstine Eriksen, Anne Tjønneland, Marie-Christine Boutron-Ruault, Joseph A. Rothwell, Gianluca Severi, Rudolf Kaaks, Verena Katzke, Matthias B. Schulze, Anna Birukov, Domenico Palli, Sabina Sieri, Maria Santucci de Magistris, Rosario Tumino, Fulvio Ricceri, Bas Bueno-de-Mesquita, Jeroen W. G. Derksen, Guri Skeie, Inger Torhild Gram, Torkjel Sandanger, J. Ramón Quirós, Leila Luján-Barroso, Maria-Jose Sánchez, Pilar Amiano, María-Dolores Chirlaque, Aurelio Barricarte Gurrea, Ingegerd Johansson, Jonas Manjer, Aurora Perez-Cornago, Elisabete Weiderpass, Marc J. Gunter, Alicia K. Heath, Casper G. Schalkwijk, Mazda Jenab

**Affiliations:** 1 Nutrition and Metabolism Section, International Agency for Research on Cancer (IARC), 69372 Lyon, France; aglagoe@fellows.iarc.fr (E.K.A.); mayenchacona@fellows.iarc.fr (A.-L.M.); freislingh@iarc.fr (H.F.); GunterM@iarc.fr (M.J.G.); 2 Early Detection, Prevention, and Infections Branch, International Agency for Research on Cancer (IARC), 69372 Lyon, France; knazev@iarc.fr; 3 Department of Epidemiology, The University of Texas MD Anderson Cancer Center, Houston, TX 77030, USA; VFedirko@mdanderson.org; 4 Cancer Biology and Therapeutics Group (CBT), Conway Institute, School of Biomolecular and Biomedical Science (SBBS), University College Dublin, D04 V1W8 Dublin, Ireland; david.hughes@ucd.ie; 5 Department of Medicine, Baylor College of Medicine, Houston, TX 77030, USA; jiao@bcm.edu; 6 Danish Cancer Society Research Center, 2100 Copenhagen, Denmark; ake@cancer.dk (A.K.E.); annet@cancer.dk (A.T.); 7 CESP, Faculté de Médecine—Université Paris-Saclay, UVSQ, INSERM, 94805 Villejuif, France; Marie-christine.BOUTRON@gustaveroussy.fr (M.-C.B.-R.); Joseph.ROTHWELL@gustaveroussy.fr (J.A.R.); Gianluca.SEVERI@gustaveroussy.fr (G.S.); 8 Gustave Roussy, 114, Rue Édouard-Vaillant, CEDEX, 94805 Villejuif, France; 9 Department of Statistics, Computer Science and Applications (DISIA), University of Florence, 50121 Florence, Italy; 10 Division of Cancer Epidemiology, German Cancer Research Center (DKFZ), 69120 Heidelberg, Germany; r.kaaks@Dkfz-Heidelberg.de (R.K.); V.Katzke@Dkfz-Heidelberg.de (V.K.); 11 Department of Molecular Epidemiology, German Institute of Human Nutrition Potsdam-Rehbruecke, 14558 Nuthetal, Germany; mschulze@dife.de (M.B.S.); Anna.Birukov@dife.de (A.B.); 12 Institute of Nutrition Science, University of Potsdam, 14558 Nuthetal, Germany; 13 Cancer Risk Factors and Life-Style Epidemiology Unit, Institute for Cancer Research, Prevention and Clinical Network (ISPRO), 50139 Florence, Italy; d.palli@ispro.toscana.it; 14 Epidemiology and Prevention Unit, Fondazione IRCCS Istituto Nazionale dei Tumori di Milano, 20133 Milan, Italy; Sabina.Sieri@istitutotumori.mi.it; 15 Azienda Ospedaliera Universitaria Federico II, 80131 Napoli, Italy; masantuc@unina.it; 16 Hyblean Association for Epidemiological Research AIRE-ONLUS, 97100 Ragusa, Italy; rtuminomail@gmail.com; 17 Department of Clinical and Biological Sciences, University of Turin, Regione Gonzole 10, 10043 Orbassano, Italy; fulvio.ricceri@unito.it; 18 Unit of Epidemiology, Regional Health Service ASL TO3, Via Sabaudia 164, 10095 Grugliasco, Italy; 19 Department for Determinants of Chronic Diseases (DCD), National Institute for Public Health and the Environment (RIVM), P.O. Box 1, 3720 BA Bilthoven, The Netherlands; basbuenodemesquita@gmail.com; 20 Julius Center for Health Sciences and Primary Care, University Medical Center Utrecht, Utrecht University, 3584 CG Utrecht, The Netherlands; H.W.G.Derksen-2@umcutrecht.nl; 21 Faculty of Health Sciences, Department of Community Medicine, University of Tromsø, The Arctic University of Norway, 9037 Tromsø, Norway; guri.skeie@uit.no (G.S.); inger.gram@uit.no (I.T.G.); torkjel.sandanger@uit.no (T.S.); 22 Public Health Directorate, 33402 Asturias, Spain; JOSERAMON.QUIROSGARCIA@asturias.org; 23 Unit of Nutrition and Cancer, Catalan Institute of Oncology—ICO; and Nutrition and Cancer Group; Epidemiology, Public Health, Cancer Prevention and Palliative Care Program, Bellvitge Biomedical Research Institute—IDIBELL, L’Hospitalet de Llobregat, Av. Granvia 199-203, 08908 Barcelona, Spain; llujan@iconcologia.net; 24 Centro de Investigación Biomédica en Red de Epidemiología y Salud Pública (CIBERESP), 28029 Madrid, Spain; mariella_san@hotmail.com (M.-J.S.); epicss-san@euskadi.eus (P.A.); mdolores.chirlaque@carm.es (M.-D.C.); aurelio.barricarte.gurrea@navarra.es (A.B.G.); 25 Escuela Andaluza de Salud Pública (EASP), 18011 Granada, Spain; 26 Instituto de Investigación Biosanitaria ibs.GRANADA, 18012 Granada, Spain; 27 Department of Preventive Medicine and Public Health, University of Granada, 18071 Granada, Spain; 28 Public Health Division of Gipuzkoa, BioDonostia Research Institute, 20014 Donostia-San Sebastian, Spain; 29 Department of Epidemiology, Murcia Regional Health Council, IMIB-Arrixaca, Murcia University, 30003 Murcia, Spain; 30 Navarra Public Health Institute, 31008 Pamplona, Spain; 31 Navarra Institute for Health Research (IdiSNA), 31008 Pamplona, Spain; 32 Department of Radiation Sciences, Oncology, Umeå University, 907 36 Umeå, Sweden; ingegerd.johansson@umu.se; 33 Department of Clinical Sciences, Malmö, Lund University, 221 00 Lund, Sweden; jonas.manjer@med.lu.se; 34 Division of Surgery, Malmö, Lund University, 221 00 Lund, Sweden; 35 Cancer Epidemiology Unit, Nuffield Department of Population Health, University of Oxford, Oxford OX3 7LF, UK; Aurora.Perez-Cornago@ndph.ox.ac.uk; 36 Office of the Director, International Agency for Research on Cancer (IARC), 69372 Lyon, France; WeiderpassE@iarc.fr; 37 Department of Epidemiology and Biostatistics, School of Public Health, Imperial College London, London W2 1PG, UK; a.heath@imperial.ac.uk; 38 Department of Internal Medicine, CARIM School for Cardiovascular Diseases, Maastricht University Medical Center, 6229 HX Maastrich, The Netherlands; c.schalkwijk@maastrichtuniversity.nl

**Keywords:** advanced glycation end-products, dietary glycation compounds, colorectal cancer, dietary exposure

## Abstract

Dietary advanced glycation end-products (dAGEs) have been hypothesized to be associated with a higher risk of colorectal cancer (CRC) by promoting inflammation, metabolic dysfunction, and oxidative stress in the colonic epithelium. However, evidence from prospective cohort studies is scarce and inconclusive. We evaluated CRC risk associated with the intake of dAGEs in the European Prospective Investigation into Cancer and Nutrition (EPIC) study. Dietary intakes of three major dAGEs: N^ε^-carboxy-methyllysine (CML), N^ε^-carboxyethyllysine (CEL), and N^δ^-(5-hydro-5-methyl-4-imidazolon-2-yl)-ornithine (MG-H1) were estimated in 450,111 participants (median follow-up = 13 years, with 6162 CRC cases) by matching to a detailed published European food composition database. Hazard ratios (HRs) and 95% confidence intervals (CIs) for the associations of dAGEs with CRC were computed using multivariable-adjusted Cox regression models. Inverse CRC risk associations were observed for CML (HR comparing extreme quintiles: HR_Q5vs_._Q1_ = 0.92, 95% CI = 0.85–1.00) and MG-H1 (HR_Q5vs_._Q1_ = 0.92, 95% CI = 0.85–1.00), but not for CEL (HR_Q5vs_._Q1_ = 0.97, 95% CI = 0.89–1.05). The associations did not differ by sex or anatomical location of the tumor. Contrary to the initial hypothesis, our findings suggest an inverse association between dAGEs and CRC risk. More research is required to verify these findings and better differentiate the role of dAGEs from that of endogenously produced AGEs and their precursor compounds in CRC development.

## 1. Introduction

Colorectal cancer (CRC) is the third most common cancer globally, and the second leading cause of cancer-related deaths [1]. The incidence of CRC follows a geographical distribution pattern, with the highest figures observed in Western countries [2], most likely attributable to the “modern” lifestyle and diet rich in energy-dense processed foods with poor nutritional value [3,4,5]. The Western diet is a substantial source of advanced glycation end-products (AGEs), an expansive group of molecules produced by irreversible non-enzymatic combination of reducing sugars and proteins, lipids, or nucleic acids [6]. The typical Western diet can also promote endogenous formation of AGEs by supplying reducing sugars and AGE precursors such as reactive dicarbonyls, i.e., methylglyoxal, glyoxal, glycolaldehyde, and glyceraldehyde [7]. Dietary AGEs (dAGEs) are known for their pro-inflammation and pro-oxidation properties in the colon and have been reported in diverse colonic pathologies, such as inflammatory bowel diseases [8]. Around 70–90% of AGEs ingested are unabsorbed [9,10] and remain in the gastrointestinal tract where they can interact directly with colon epithelial cells. The human colon is, therefore, potentially exposed to AGEs from the diet, but also from the systemic milieu by way of circulating AGEs [11,12].

AGEs have been hypothesized to be associated with CRC development [13], mostly due to their ability to promote tumor cell growth in vitro [13]. A body of mechanistic evidence has linked AGEs to CRC through stimulation of the pro-inflammatory response via the activation of the receptor of AGEs (RAGE) [14], an increase in colonic barrier permeability—allowing closer interaction of AGEs with colonic epithelium—and consequential leakage of bacterial toxins into the systemic circulation [15]. Notwithstanding these numerous plausible mechanisms, no previous epidemiological studies have investigated the relationship between dAGEs and CRC, probably due to the lack of detailed food composition databases for these compounds. The development of food composition tables for estimating dietary AGEs is recent, and few tables exist, mainly for Japanese foods [16] and more recently for European foods [17]. Due to the large number of different AGEs, the tables developed focused on the major compounds, specifically N^ε^-(carboxymethyl)lysine (CML). The European food composition table provides data on CML and, additionally, on two other major dAGEs: N^ε^-(carboxyethyl)lysine (CEL) and N^δ^-(5-hydro-5-methyl-4-imidazolon-2-yl)-ornithine (MG-H1).

Considering the potential direct interaction of dAGEs with the colonic epithelium and their numerous CRC-promoting effects, we hypothesize a positive CRC risk association with higher dAGEs consumption. We evaluated our hypothesis using information on dietary intake of CML, CEL, and MG-H1 in the prospective European Prospective Investigation into Cancer and Nutrition (EPIC) cohort.

## 2. Materials and Methods

### 2.1. Study Participants

We used data from the EPIC study, a large prospective cohort with over half a million participants (*n* = 521,324) from 10 European countries (Denmark, France, Germany, Greece, Italy, the Netherlands, Norway, Spain, Sweden, and the United Kingdom) [18]. In brief, participants aged between 35 and 75 years were recruited from 1992 to 2000 in 23 participating centers. Anthropometric measures, socio-demographic information, and lifestyle and dietary intake data were collected at recruitment from all participants. Standing height, weight, and waist and hip circumferences were measured, with self-reporting exceptions in France, Norway, and Oxford. Body mass index (BMI, in kg/m^2^) was calculated.

### 2.2. Ethical Considerations

Ethical approval for the EPIC study was obtained from the Ethical Committee of the International Agency for Research on Cancer (IARC) and local ethical committees. All participants provided written consent to participate in the study.

### 2.3. Dietary Assessment and dAGEs Estimation

Usual diet was collected at baseline using a combination of country- or center-specific questionnaires that have been validated to reflect local contexts [19,20]. Dietary data were collected during interviews in Greece, Spain, and Naples and Ragusa (Italy), whereas in other EPIC centers self-administrated questionnaires were used. Quantitative dietary questionnaires were used in Germany, Greece, the Netherlands, and Northern Italy; semi-quantitative food frequency questionnaires were employed in Denmark, Norway, Naples, Umea, and the UK; and in Malmo a combination of a non-quantitative food-frequency questionnaire and a food record was utilized.

To estimate intakes of individual AGEs, we used the database for protein-bound AGEs developed for 190 food items selected from the Dutch cohort of EPIC and the Dutch National Food Consumption survey [17]. These foods were matched to the EPIC food list by name and descriptors, especially considering preparation and processing to expand the EPIC Nutrient Database (ENDB) with extra food components, a procedure used for other nutrients/anti-nutrients and described in detail elsewhere [21,22]. For complex foods with multiple ingredients, the foods were decomposed into specific ingredients or food items to generate EPIC dAGEs composition data for each food item. Thereafter, for each participant, daily intakes of CML, CEL, and MG-H1 were estimated. ∑dAGEs was calculated as the sum of individual dAGEs (CML+CEL+MG-H1) and used to picture overall AGEs intake patterns. The main food contributors of dietary CML and MG-H1 were (from highest to lowest contribution): cereals and cereal products, meats and meat products, cakes, and biscuits (Supplementary Figure S1) [23]. For CEL, the main contributors were meat and meat products, cereal and cereal products, cakes, and biscuits. Dairy products, fish and fish products, and non-alcoholic drinks were also relevant dietary sources of the three dAGEs.

We excluded participants from Greece (*n* = 26,048) due to data use restrictions, those diagnosed with cancer at baseline (*n* = 25,184), those with missing follow-up information (*n* = 4148) or dietary questionnaire data (*n* = 6259), those in the highest or lowest 1% of energy intake versus energy requirements (*n* = 9573), and a participant who withdrew from EPIC. Our final dataset included 450,111 participants, among whom 318,686 were women (71%).

### 2.4. Identification of CRC Cases

Cancer cases were ascertained from cancer registries in Denmark, Italy, the Netherlands, Norway, Spain, Sweden, and the United Kingdom, or by using a combination of sources, including health insurance records, oncology and pathology records, or, in the specific cases of France and Germany, through active follow-up of the participants and their relatives. CRC cases were first incident, and histologically confirmed by a pathologist. We used the International Classification of Diseases for Oncology (ICD-O, codes C18–C20) to define the cases. Colon cancers were defined as tumors that occurred in the cecum, appendix, ascending colon, hepatic flexure, transverse colon, splenic flexure, or the descending or sigmoid colon (C18.0–C18.7), and overlapping and/or unspecified origin tumors (C18.8 and C18.9). Rectal cancers were defined as tumors that occurred at the recto-sigmoid junction (C19) or rectum (C20).

### 2.5. Statistical Analyses

Daily intakes of CML, CEL, and MG-H1 were natural log-transformed and their standardized residuals were computed by regressing the ln-transformed values on participant energy intake and center. Ln-transformed dAGEs were divided into quintiles, with the first quintile used as the reference in all our analyses. Cox proportional hazards regression models stratified by age at recruitment (one-year categories), sex, and center were used to compute hazard ratios (HRs) and 95% confidence intervals (CIs) for the association between individual dAGEs and CRC risk. Time at entry was age at recruitment, while exit time was set as the age at which any of the following first occurred: CRC diagnosis, death, emigration, or last date at which follow-up was considered complete. To test the trends of the associations, we ran Cox models using median values of each category as a continuous variable. Analyses were also conducted using continuous variables for dAGEs (per ln(SD) increment). No deviation from the proportional hazards assumption was observed after assessing Schoenfeld residuals. Three main models were run. Model 1 was stratified by age (1-year categories), sex, and center. Model 2 was additionally adjusted for BMI (continuous), height (continuous), and lifestyle factors, including education (none; primary; technical and professional; secondary, higher), physical activity (inactive; moderately inactive; moderately active; active), smoking status and intensity (never; current smokers, cigarettes/day: 1–15, 16–25, >26; former smokers who quit: <=10, 11–20, >20 years; occasional), and total energy intake (kcal/day, continuous). Model 3 was further adjusted for the Mediterranean diet score to consider the diet as a whole, and because this score has also been specifically associated with CRC risk [24]. We considered missing data as a separate category for physical activity (1.9%), education (2.3%), and smoking (3.3%). Restricted cubic splines were used to model possible nonlinear trends [25,26]. Linearity of the associations was tested using the likelihood ratio test, comparing the model with only the linear term with the model including both the linear and the cubic spline terms. There was no indication of nonlinear associations in any of our analyses. Analyses (using Model 3) by anatomical subsites of the colorectum were also run for rectal and colon cancer, and, specifically, for proximal and distal colon cancers. Potential differences in the associations by tumor sites, i.e., rectal vs. colon or proximal colon vs. distal colon, were tested using competing risk analyses [27,28]. Stratified analyses by country, BMI, sex, and years of follow-up were conducted and multiplicative interactions were included in the model to evaluate potential heterogeneity. To model the possible impact of reverse causation, we ran sensitivity analyses by excluding the first 2 years of follow-up.

All the analyses were carried out using Stata 14.0 (StataCorp., College Station, TX, USA). We considered two-sided *p*-values below 0.05 as statistically significant.

## 3. Results

Table 1 summarizes selected baseline characteristics of the study participants by quintiles of ∑dAGEs. Participants in the highest quintile consumed more processed meats, cakes, biscuits, cereals and cereal products, and legumes and less fruit. They also tended to consume less sugar, confectionery, and alcohol.

In minimally adjusted models (Model 1), CML (HR comparing highest to lowest quintile, HR_Q5vs_._Q1_ = 0.83, 95% CI = 0.77–0.90, *p* for trend <0.001) and MG-H1 (HR_Q5vs_._Q1_ = 0.84, 95% CI = 0.77–0.90, *p* for trend <0.001) showed inverse associations with CRC, whereas no significant association was observed for CEL (HR_Q5vs_._Q1_ = 0.92, 95% CI = 0.85–1.00, *p* for trend = 0.064) (Table 2). The significance of the associations was attenuated after full adjustment (Model 3), with HR (95% CI) of 0.97 (0.94–0.99) and 0.97 (0.95–1.00) for CML and MG-H1, respectively.

Analyses by tumor subsites showed no significant heterogeneity between colon and rectal cancers, although the association with rectal cancer was statistically significant for CML (HR per ln(SD) increment: rectal cancer HR_ln(SD)_ = 0.93, 95% CI = 0.88–0.97) and MG-H1 (rectal cancer HR_ln(SD)_ = 0.94, 95% CI = 0.90–0.99) (Table 3). No significant difference in the association between the individual dAGEs and CRC risk was observed by sex (Supplementary Table S1). In stratified analyses, dAGEs–CRC risk did not differ by country, and tended to be restricted to participants with BMI <30 kg/m^2^ (Supplementary Table S2). When the follow-up time of cases was considered, dAGEs–CRC showed a gradient in the association between higher CRC risk observed and lower follow-up. Excluding participants with follow-up less than 2 years did not materially change the results (not shown).

## 4. Discussion

In this large prospective study, we found that dietary intakes of CML and MG-H1, but not CEL, were inversely associated with the risk of CRC. Our analyses did not identify any heterogeneity in these findings by anatomical subsite of the tumor within the colorectum, by sex, or by follow-up time.

Our findings were contrary to our initial hypothesis that dAGE exposure could promote CRC development. This hypothesis was based on considerable experimental evidence suggesting cancer-promoting characteristics for these compounds. Three main mechanisms have been postulated: first, AGEs may bind to the RAGE receptor in colonocytes and, subsequently, promote and sustain inflammation and oxidative stress [29,30]; second, they may modify the composition of the microbiome towards microbial genera that are deleterious to gut health [31]; and finally, they may increase gut permeability, thereby allowing bacterial translocation and increased exposure of colonocytes to toxic bacterial compounds [32]. In in vitro enterocyte models, cells treated with AGEs have shown higher expression of RAGE and an increase in inflammatory factors, such as IL-8, IL-1β, and nuclear factor-kappa B (NF-κB) [13,33], suggesting that dAGEs may produce similar effects in the gut. Nevertheless, a main condition for this to occur in vivo is that CML, CEL, and MG-H1 need to reach the colon untransformed, in a protein-bound form which could be recognized by RAGE and be able to interact with the cell surface of colonocytes. This is because several studies have reported that free AGEs or those attached to single amino acids are not as recognizable by RAGE as protein-bound AGEs [34,35]. A recent study using a dynamic in vitro model showed that protein-bound dAGEs can survive intestinal digestion and remain in the gastrointestinal tract [36]. Zenker et al. [37], using a model with casein, have shown in a recent study that unglycated proteins could also interact with RAGE. This provides additional evidence supporting the complexity of the AGEs, particularly their interactions in the gastrointestinal milieu. It is evident that more knowledge is needed on the role of the microbiome and intestinal conditions in the conservation or degradation of protein-bound dAGEs, and how this may affect RAGE-specific inflammation.

Recent growing evidence suggested that the human gut microbiome can metabolize dAGEs, possibly as much as 40% for ingested CML [38]. CML has been shown to be metabolized by the microbiome into several sub-products, including biogenic amines and fatty acids, notably N-carboxymethylcadaverine, N-carboxymethylaminopentanoic acid, N-carboxymethyl-Δ1-piperideinium ion, and 2-amino-6-(formylmethylamino)hexanoic acid [39,40]. Less is known about the specific actions of these catabolic products within the colorectum, or the possible downstream molecules that can be produced from them. It is possible that these compounds may not be recognized by RAGE, and, hence, not induce an inflammatory response within the gut. Nevertheless, dAGEs have been associated with reduced diversity and richness of the gut microbiome, which is thought to be conducive to a CRC-promoting environment [15]. Thus, the possible microbial metabolism of dAGEs may not entirely negate their deleterious properties. It is also noteworthy that the three dAGEs that we assessed are thought to bind to a single domain of RAGE (V domain), whereas other AGE compounds, such as pentosidine, which are much less abundant in the diet, could bind to additional domains (V and C1 domains) [41], potentially triggering a stronger inflammatory response. This suggests that future studies should also consider studying the potential deleterious properties of other less abundant dAGEs and their possible CRC risk associations.

We are unsure why we observed inverse CRC risk associations with CML and MG-H1, whereas CEL demonstrated no association. One possible explanation may relate to the different dietary sources for these compounds. In EPIC, CML and MG-H1 share very similar food sources (e.g., mostly cereals and cereal products), while CEL is derived to a greater extent from meats [23,42]. Cereals and cereal products are major sources of dietary fiber, which has been previously associated with lower CRC risk and could partially explain the inverse association observed with CML and MG-H1 [43]. Another potential explanation for the differential CRC risk associations for these compounds may be the chemical pathways through which they are produced. Although the three AGEs are all likely derived from reducing sugars, their main precursors are reactive dicarbonyl compounds, particularly glyoxal (GO) and methylglyoxal (MGO). CML originates from GO, whereas CEL and MG-H1 are mainly produced from MGO [44]. In addition, CEL and MG-H1 (the two MGO-derived AGEs) differ in their amino acid content, as CEL is produced from lysine, while MG-H1 derives from arginine [45]. Interestingly, reactive dicarbonyl compounds are thought to possess a glycating potential that may be thousands of times higher compared to that of sugars such as glucose or fructose [46] and, hence, the CRC risk association of these dietary compounds and their precursors may also warrant further study.

The importance of studying dAGEs in CRC development lies in several potential public health measures that may be taken to control their exposures at wider population levels. For example, dAGEs may be directly targeted through adoption of specific cooking methods (e.g., steaming) to reduce the dAGE content of specific processed foods, or possibly with anti-glycation dietary compounds (e.g., polyphenols) [47] to counter any possible adverse effects [48,49].

Although, to date, no large prospective studies have explored the association between dAGEs and CRC development, the role of circulating AGEs in CRC development has been explored in three separate case-control studies nested within prospective cohorts, with discordant findings. Two of the three studies reported inverse associations with CRC [50,51], whereas one reported a positive association in male smokers [52]. It is noteworthy that, among these previous studies, two [50,52] estimated AGEs using ELISA, which is now recognized as providing biased AGE estimation [53]. Nevertheless, taken together with our study, these observations suggest that the role of AGEs in CRC development is likely to be quite complex, and further studies of other AGE compounds beyond the three studied here are also warranted.

The strengths of our study include its large study population, its prospective design, and the large number of CRC cases, which allowed us to run extensive analysis and control for a comprehensive number of confounders. In addition, our study followed the recent “quality control” recommendations for studies on AGEs, i.e., the study of several specific AGEs and the use of a validated food composition database to estimate individual dAGE exposures [54]. However, our study was limited by the single collection of dAGEs and other covariates at baseline; thus, potential changes in diet or covariates during follow-up could not be accounted for. AGE levels in the foods are influenced by cooking methods, i.e., frying, baking, or broiling, and conditions, such as cooking temperature, humidity, and pH [55]. Hence, it is possible that country-specific differences in cooking conditions and varying geographical and/or individual preferences for doneness of similar foods items could have impacted our dietary AGE estimation. Additionally, it is possible that residual confounding cannot be completely ruled out.

In conclusion, we found inverse associations between the intake of CML and of MG-H1, but not of CEL, and the risk of CRC in the large EPIC prospective cohort. Our findings corroborate some previous findings from circulating AGEs, suggesting that the three AGEs included in our study may not be CRC-promotive, as has previously been suspected. Our study provides additional evidence of the complexity of AGEs and their interaction in CRC and calls for additional studies to confirm our findings and to explore the link between CRC and other dAGEs not studied herein.

## Figures and Tables

**Table 1:** Selected characteristics of the study participants (by quintiles of dietary AGEs), EPIC cohort study, 1992–2014.

	Quintiles of ∑AGEs Intake				
	Quintile 1	Quintile 2	Quintile 3	Quintile 4	Quintile 5
**Recruitment and follow-up**					
Age at recruitment, years	50.9 ± 9.9	50.5 ± 9.7	50.2 ± 9.7	50.1 ± 9.7	51.5 ± 9.7
Follow-up, years	14.1 ± 4.2	14.1 ± 4	14.1 ± 3.9	14.1 ± 3.8	14.2 ± 4.1
**Anthropometry**					
BMI, kg/m^2^	25.2 ± 4.2	25.3 ± 4.2	25.3 ± 4.2	25.3 ± 4.2	25.1 ± 4.2
Socio-demographic and lifestyle *					
Education status, %					
None	3.3	3.5	3.5	3.7	3.6
Primary school	25.5	24.9	24.9	24.8	25.0
Technical or professional	23.0	24.0	23.9	23.3	22.7
Secondary school	20.6	20.6	21.3	22.3	21.0
Higher education	24.7	24.4	24.3	24.2	25.1
Smoking status, %					
Never	38.2	41.4	43.0	44.1	46.0
Current, 1–<16 cigarettes/day	12.8	12.3	11.9	11.5	9.58
Current, 16–<=20 cigarettes/day	8.1	6.75	6.1	5.5	4.19
Current, >20 cigarettes/day	2.4	1.57	1.3	1.2	0.88
Former, quit <=10 years	9.7	9.89	9.7	9.7	9.15
Former, quit 11–<20 years	8.1	8.42	8.4	8.4	8.56
Former, quit >20 years	7.8	7.83	8.1	8.0	9.22
Current, pipe-cigar-occasional	9.5	8.46	8.5	8.6	9.31
Physical activity status, %					
Inactive	21.2	19.8	19.2	18.6	19.0
Moderately inactive	33.3	33.7	33.3	32.9	33.4
Moderately active	25.2	26.7	27.0	27.5	27.0
Active	18.5	18.0	18.4	18.5	19.0
**Daily dietary intake**					
Energy intake, kcal	2052 ± 775	2084 ± 639	2091 ± 585	2092 ± 548	2063 ± 512
Red meat, g	44.4 ± 40.3	44.6 ± 37	43.3 ± 35.2	42.0 ± 34.1	38.9 ± 33.1
Processed meat, g	30.6 ± 30.7	33.1 ± 30	34.1 ± 30.2	34.1 ± 30.1	34.7 ± 32.6
Fibre, g	20.1 ± 8.1	22.0 ± 7.4	23.1 ± 7.2	23.9 ± 7.3	25.1 ± 7.9
Dairy products, g	345 ± 271	332 ± 237	326 ± 229	324 ± 224	341 ± 226
Fish and shellfish, g	36.7 ± 37.2	38.6 ± 37.1	38.4 ± 36.7	37.6 ± 35.9	38.2 ± 34.7
Cakes and biscuits, g	29.0 ± 34.9	39.3 ± 40.5	45.0 ± 43.4	49.2 ± 45.8	47.9 ± 46.5
Cereal and cereal products, g	171 ± 101	207 ± 105	224 ± 106	237 ± 108	260 ± 121
Fruits, nuts, and seeds, g	242 ± 220	235 ± 183	231 ± 169	228 ± 160	225 ± 156
Vegetables, g	205 ± 146	198 ± 127	193 ± 122	190 ± 121	200 ± 128
Legumes, g	10.2 ± 18.7	13.2 ± 21.7	14.8 ± 23.7	16.2 ± 25.7	16.8 ± 27.2
Potatoes and other tubers, g	100 ± 86.3	96 ± 75.1	92.0 ± 69.8	89 ± 68.3	93 ± 69.2
Egg and egg products, g	17.6 ± 18.7	18.2 ± 17	18.2 ± 16.5	18.1 ± 16.6	17.8 ± 17.1
Fat, g	79.9 ± 35.5	81.3 ± 29.8	81.1 ± 27.6	80.6 ± 26.5	78.4 ± 25.9
Sugar and confectionery, g	50.3 ± 76.3	44.3 ± 45.4	41.8 ± 38.7	39.8 ± 36.2	37.6 ± 33.4
Alcohol, g	18.9 ± 25	13.1 ± 16.5	10.9 ± 14.1	9.3 ± 12.5	8.3 ± 11.3
Mediterranean diet score, %					
Low	32.8	26.2	23.9	22.8	21.2
Medium	44.6	46.5	47.1	47.9	49.3
High	22.6	27.3	28.9	29.3	29.6

Abbreviation: AGE, advanced glycation end-products; EPIC, European Prospective Investigation into Cancer and Nutrition; mean ± standard deviation is presented, unless otherwise stated; quintiles were calculated using total energy, center-standardized residuals, and log-transformed ∑AGEs values. * Percentages do not add up to 100% because of missing values.

**Table 2:** Hazard ratios and 95% confidence intervals (CI) for colorectal cancer risk associated with individual dietary AGEs (quintiles and continuous), EPIC cohort study, 1992–2014.

Dietary AGE	*N* Cases	Median Intake	Model 1	Model 2	Model 3
CML, mg/day					
Quintile 1	1391	1.90	1.00 (Ref.)	1.00 (Ref.)	1.00 (Ref.)
Quintile 2	1259	2.41	0.93 (0.86–1.00)	0.94 (0.87–1.02)	0.98 (0.90–1.06)
Quintile 3	1210	2.75	0.91 (0.85–0.99)	0.94 (0.87–1.01)	0.98 (0.91–1.07)
Quintile 4	1120	3.16	0.85 (0.79–0.92)	0.88 (0.81–0.95)	0.93 (0.86–1.01)
Quintile 5	1182	4.02	0.83 (0.77–0.90)	0.87 (0.80–0.94)	0.92 (0.85–1.00)
*p* for trend			<0.001	<0.001	0.023
per ln(SD) increase			0.94 (0.91–0.96)	0.95 (0.92–0.97)	0.97 (0.94–0.99)
CEL, mg/day					
Quintile 1	1214	1.37	1.00 (Ref.)	1.00 (Ref.)	1.00 (Ref.)
Quintile 2	1271	1.71	1.00 (0.92–1.08)	1.00 (0.92–1.08)	1.02 (0.95–1.11)
Quintile 3	1219	1.93	0.95 (0.88–1.03)	0.95 (0.88–1.03)	0.99 (0.91–1.07)
Quintile 4	1268	2.21	0.99 (0.91–1.07)	1.00 (0.92–1.08)	1.04 (0.96–1.13)
Quintile 5	1190	2.85	0.92 (0.85–1.00)	0.92 (0.85–1.00)	0.97 (0.89–1.05)
*p* for trend			0.064	0.072	0.630
per ln(SD) increase			0.97 (0.95–1.00)	0.97 (0.95–1.00)	0.99 (0.96–1.01)
MG-H1, mg/day					
Quintile 1	1388	13.0	1.00 (Ref.)	1.00 (Ref.)	1.00 (Ref.)
Quintile 2	1250	16.7	0.94 (0.87–1.01)	0.94 (0.87–1.02)	0.98 (0.90–1.06)
Quintile 3	1183	19.3	0.91 (0.84–0.99)	0.93 (0.86–1.00)	0.97 (0.90–1.06)
Quintile 4	1120	22.5	0.88 (0.81–0.95)	0.89 (0.82–0.97)	0.94 (0.87–1.03)
Quintile 5	1221	29.9	0.84 (0.77–0.90)	0.86 (0.80–0.94)	0.92 (0.85–1.00)
*p* for trend			<0.001	<0.001	0.033
per ln(SD) increase			0.94 (0.92–0.97)	0.95 (0.93–0.98)	0.97 (0.95–1.00)

Abbreviations: AGE, advanced glycation end-product; CML, Nε-carboxy-methyllysine; CEL, Nε-carboxy-ethyllysine; EPIC, European Prospective Investigation into Cancer and Nutrition; MG-H1, Nδ-(5-hydro-5-methyl-4-imidazolon-2-yl)-ornithine; Model 1 is stratified by age (1-year categories), sex, and center; Model 2 is additionally adjusted for BMI, height, education, physical activity, smoking, and energy intake; Model 3 is Model 2 additionally adjusted for the Mediterranean diet score.

**Table 3:** Hazard ratios and 95% confidence intervals (CI) for the risk of colorectal cancer in anatomical subsites associated with dietary AGEs (quintiles and continuous), EPIC cohort study, 1992–2014.

	Median Intake	Colon Cancer						Rectal Cancer	
		All		Proximal Colon		Distal Colon			
		*N* Cases	HR (95% CI )	*N* Cases	HR (95% CI )	*N* Cases	HR (95% CI )	*N* Cases	HR (95% CI )
CML, mg/day									
Quintile 1	1.90	873	1.00 (Ref.)	399	1.00 (Ref.)	397	1.00 (Ref.)	518	1.00 (Ref.)
Quintile 2	2.41	774	0.95 (0.86–1.05)	357	0.95 (0.82–1.10)	317	0.87 (0.75–1.02)	485	1.02 (0.90–1.17)
Quintile 3	2.75	786	1.01 (0.91–1.11)	366	1.01 (0.87–1.17)	319	0.92 (0.79–1.08)	424	0.95 (0.83–1.09)
Quintile 4	3.16	759	0.99 (0.89–1.09)	345	0.95 (0.82–1.11)	351	1.04 (0.89–1.21)	361	0.81 (0.70–0.94)
Quintile 5	4.02	805	0.98 (0.89–1.09)	389	0.99 (0.85–1.15)	342	0.97 (0.83–1.13)	377	0.81 (0.70–0.93)
*p* for trend			0.989		0.944		0.564		<0.001
per ln(SD) increase			0.99 (0.96–1.02)		1.00 (0.95–1.05)		0.99 (0.94–1.04)		0.93 (0.88–0.97)
CEL, mg/day									
Quintile 1	1.37	763	1.00 (Ref.)	336	1.00 (Ref.)	357	1.00 (Ref.)	451	1.00 (Ref.)
Quintile 2	1.71	830	1.06 (0.95–1.17)	380	1.10 (0.94–1.27)	365	1.00 (0.86–1.16)	441	0.99 (0.86–1.13)
Quintile 3	1.93	789	1.01 (0.91–1.12)	348	1.02 (0.87–1.19)	353	0.98 (0.84–1.14)	430	0.94 (0.82–1.08)
Quintile 4	2.21	828	1.08 (0.97–1.19)	402	1.18 (1.01–1.37)	323	0.92 (0.79–1.07)	440	0.99 (0.86–1.14)
Quintile 5	2.85	787	1.01 (0.91–1.12)	390	1.12 (0.96–1.31)	328	0.93 (0.79–1.08)	403	0.89 (0.77–1.03)
*p* for trend			0.697		0.083		0.194		0.166
per ln(SD) increase			1.00 (0.97–1.04)		1.03 (0.99–1.09)		0.97 (0.92–1.02)		0.96 (0.92–1.00)
MG-H1, mg/day									
Quintile 1	13.0	861	1.00 (Ref.)	384	1.00 (Ref.)	386	1.00 (Ref.)	527	1.00 (Ref.)
Quintile 2	16.7	806	1.00 (0.91–1.10)	367	1.02 (0.88–1.19)	352	0.98 (0.84–1.13)	444	0.95 (0.83–1.09)
Quintile 3	19.3	770	1.01 (0.91–1.11)	351	1.02 (0.87–1.18)	342	1.01 (0.86–1.17)	413	0.93 (0.81–1.07)
Quintile 4	22.5	735	0.98 (0.89–1.09)	348	1.03 (0.89–1.20)	306	0.92 (0.78–1.08)	385	0.89 (0.77–1.02)
Quintile 5	29.9	825	0.99 (0.90–1.10)	406	1.06 (0.91–1.23)	340	0.94 (0.81–1.10)	396	0.81 (0.71–0.94)
*p* for trend			0.793		0.457		0.326		0.003
per ln(SD) increase			0.99 (0.96–1.02)		1.03 (0.98–1.07)		0.96 (0.92–1.01)		0.94 (0.90–0.99)

Abbreviations: AGE, advanced glycation end-product; CI, confidence interval; CML, Nε-carboxy-methyllysine; CEL, Nε-carboxy-ethyllysine; EPIC, European Prospective Investigation into Cancer and Nutrition; HR, hazard ratio; MG-H1, Nδ-(5-hydro-5-methyl-4-imidazolon-2-yl)-ornithine; models were adjusted for body mass index, height, education, physical activity, smoking, energy intake and Mediterranean diet score and stratified by age (1-year categories), sex, and center; *p* for heterogeneity between colon and rectal cancer was 0.391, 0.849, and 0.825 for CML, CEL, and MG-H1, respectively; *p* for heterogeneity between proximal and distal colon cancer was 0.878, 0.793, and 0.804 for CML, CEL, and MG-H1, respectively.

## Data Availability

Data described in the study, code book and analytic code will be made available upon request. For information on how to submit an application for gaining access to EPIC data and/or biospecimens, please follow the instructions at http://epic.iarc.fr/access/index.php.

## Supplementary Materials

The following are available online at https://www.mdpi.com/article/10.3390/nu13093132/s1, Supplementary Table S1: Hazard ratios and 95% confidence intervals (CI) for colorectal cancer risk associated with AGEs intake, by sex, EPIC cohort study, 1992–2014; Supplementary Table S2: Stratified analysis for colorectal cancer risk associated with AGEs intake by BMI, years of follow-up and by EPIC country; Supplementary Figure S1: Dietary contribution of individual advanced glycation end-products in the European Prospective Investigation into Cancer and Nutrition (EPIC).

## Abbreviations

AGE: advanced glycation end-product; BMI, body mass index; CEL, N^ε^-(carboxyethyl)lysine; CI, confidence interval; CML, N^ε^-(carboxymethyl)lysine; CRC, colorectal cancer; EPIC, European Prospective Investigation into Cancer and Nutrition; IARC, International Agency for Research on Cancer; ICD, international classification of diseases; MG-H1, Nδ-(5-hydro-5-methyl-4-imidazolon-2-yl)-ornithine; RAGE, receptor for AGE; SD, standard deviation.
