# Supplementary material for: Dietary Advanced Glycation End-Products and Colorectal Cancer Risk in the European Prospective Investigation into Cancer and Nutrition (EPIC) Study
Source: Nutrients. 2021 Sep 8;13(9):3132. doi: 10.3390/nu13093132 (PMC8470201; doi:10.3390/nu13093132)
Supplement: Supplementary file 1 [file nutrients-13-03132-s001.zip › nutrients-1356582-supplementary.pdf]

Supplementary Table S1: Hazard ratios and 95% confidence intervals (CI) for colorectal cancer risk associated with AGEs intake, by sex, EPIC cohort study, 1992-2014

	**Men (n=2,651 cases)**		Women (n=3,511 cases)		*P* for heterogeneity
	**Mean intake**	HR (95%CI)	Mean intake	HR (95%CI)	
CML, mg/day	3.54±1.42	0.97 (0.93 - 1.01)	2.9±1.2	0.96 (0.93 - 1.00)	*0.971*
CEL, mg/day	2.51±1.04	0.99 (0.95 - 1.03)	2.04±0.85	0.98 (0.95 - 1.02)	*0.600*
MG-H1, mg/day	24.3±10.9	0.97 (0.93 - 1.01)	20.6±9	0.98 (0.94 - 1.01)	*0.667*

Abbreviations: AGE, Advanced Glycation End-product; CML, Nε-carboxy-methyllysine; CEL, Nε-carboxy-ethyllysine; MG-H1, Nδ-(5-hydro-5-methyl-4-imidazolon-2-yl)-ornithine. Models were adjusted for BMI, height, education, physical activity, smoking, energy intake, Mediterranean diet score and stratified by age (1-year categories) and centre.

Supplementary Table S2: Stratified analysis for colorectal cancer risk associated with AGEs intake by BMI, years of follow-up and by EPIC country.

		**CML, mg/day**	**CEL, mg/day**	**MG-H1, mg/day**
	N cases	HR (95%CI)	HR (95%CI)	HR (95%CI)
**BMI**				
<25 kg/m²	2,575	0.96 (0.92 - 1.00)	0.99 (0.95 - 1.03)	0.98 (0.94 - 1.02)
25-<30kg/m²	2,578	0.97 (0.93 - 1.01)	0.95 (0.91 - 1.00)	0.96 (0.92 - 1.00)
>30 kg/m²	1,009	1.02 (0.95 - 1.09)	1.01 (0.94 - 1.07)	1.01 (0.94 - 1.08)
P for heterogeneity		*0.047*	*0.141*	*0.057*
**Years of follow-up for cases**				
<=3 years	1,041	1.05 (0.98 - 1.13)	1.01 (0.93 - 1.09)	1.03 (0.96 - 1.10)
>3&<=10 years	2,683	1.00 (0.96 - 1.04)	0.98 (0.94 - 1.03)	1.00 (0.96 - 1.04)
>10 years	2,438	0.97 (0.93 - 1.01)	0.99 (0.95 - 1.04)	0.98 (0.94 - 1.02)
P for heterogeneity		*0.018*	*0.919*	*0.027*
**Country**				
France	418	0.98 (0.88 - 1.08)	1.02 (0.92 - 1.12)	1.03 (0.93 - 1.14)
Italy	588	0.99 (0.89 - 1.10)	1.04 (0.93 - 1.17)	1.01 (0.90 - 1.12)
Spain	568	0.98 (0.89 - 1.08)	0.98 (0.91 - 1.06)	0.96 (0.87 - 1.05)
UK	1,024	0.95 (0.90 - 1.01)	0.94 (0.88 - 1.00)	0.96 (0.90 - 1.02)
Netherlands	517	1.00 (0.89 - 1.14)	1.04 (0.92 - 1.17)	0.96 (0.84 - 1.09)
Germany	461	0.99 (0.89 - 1.11)	0.98 (0.89 - 1.09)	0.98 (0.88 - 1.09)
Sweden	933	0.96 (0.90 - 1.02)	0.97 (0.91 - 1.03)	0.98 (0.93 - 1.04)
Denmark	1,342	0.98 (0.91 - 1.04)	0.92 (0.85 - 0.99)	0.95 (0.89 - 1.01)
Norway	311	0.96 (0.83 - 1.10)	0.95 (0.80 - 1.13)	1.01 (0.87 - 1.16)
P for heterogeneity		*0.907*	*0.428*	*0.868*

Abbreviations: AGE, Advanced Glycation End-product; CML, Nε-carboxy-methyllysine; CEL, Nε-carboxy-ethyllysine; MG-H1, Nδ-(5-hydro-5-methyl-4-imidazolon-2-yl)-ornithineModels were adjusted for BMI (except for model for BMI), height, education, physical activity, smoking, energy intake, Mediterranean diet score and stratified by age (1-year categories) and centre (except for model for Country)

Supplementary Figure S1: Dietary contribution of individual advanced glycation end-products in the European Prospective Investigation into Cancer and Nutrition (EPIC).

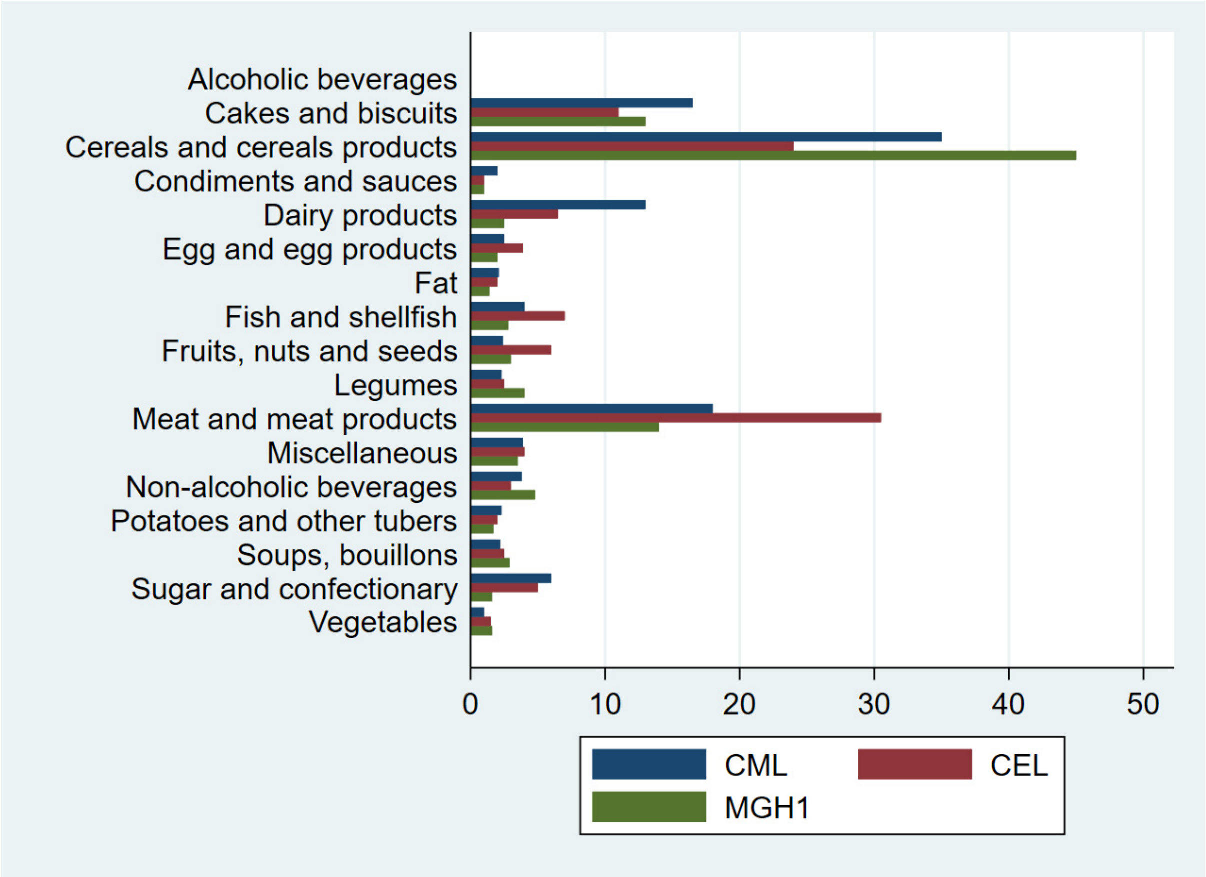

Abbreviations: AGE, Advanced Glycation End-product; CML, Nε-carboxy-methyllysine; CEL, Nε-carboxy-ethyllysine; MG-H1, Nδ-(5-hydro-5-methyl-4-imidazolon-2-yl)-ornithine
